# Editorial: Immunological processes in maxillofacial bone pathology

**DOI:** 10.3389/fimmu.2024.1394835

**Published:** 2024-03-13

**Authors:** Matthias Tröltzsch

**Affiliations:** ^1^ Center for Oral, Maxillofacial and Facial Reconstructive Surgery, Ansbach, Germany; ^2^ Department of Oral and Maxillofacial Surgery and Facial Plastic Surgery, University Hospital, LMU Munich, Munich, Germany

**Keywords:** immunology, periodontitis, MRONJ, dry socket (alveolitis osteitis), periimplantitis, romosozumab

The importance of immunological processes in maxillofacial bone pathologies may not seem obvious to many oral health care providers. For many of us, the “jaw” consists mainly of the dentition, gingiva, and oral mucosa. The bone is taken for granted and considered an inert and reliable tissue. Bone anatomy, physiology, and pathology are taught extensively in graduate and postgraduate training programs. Students and trainees get the impression that bone pathology is equivalent to odontogenic tumors, tumor-like lesions, and neoplasia, the treatment of which is limited to specialists (1). Therefore, clinicians may have limited interest in a Research Topic about “*immunological processes in maxillofacial bone pathologies*”.

The truth is that a thorough understanding of physiological and biochemical processes within the jawbone is essential to provide superb clinical care. Maxillofacial bone pathology is omnipresent: periodontitis, periimplantitis, dry socket, osteomyelitis, medication-related osteonecrosis of the jaw (MRONJ), arthritis of the temporomandibular joint – just to name a few “mundane” diseases that are caused and maintained by immunological processes (2–4). Causative treatment of those diseases is only possible if their pathophysiology is understood which is the link between research and practice (translational medicine). For example, periimplantitis is caused by a complex host response to bacterial periimplant colonization (5). The causative treatment of periimplantitis could either be achieved by host response modulation (6) and/or bacterial elimination (7). While host response modulation therapies are still experimental the only treatment option is surface decontamination which can be achieved by mechanical and physicochemic methods (7).

MRONJ is triggered by decreased defense capacities of the jawbone due to antiresorptive-drug-induced immune suppression, acidification, and osteoclast inhibition (2). While many factors have been investigated and discussed, the inflammatory stimulus (immunological process) is the decisive factor for the onset and maintenance of MRONJ is unquestionably the decisive step (8). If the inflammatory trigger can be prevented or eliminated, MRONJ can be avoided and controlled.

Immunology is an important link between intraoral and systemic diseases. Rheumatic diseases such as rheumatoid arthritis can be triggered and/or maintained by inflammatory oral processes such as periodontitis (Krutyholowa et al.). Studies have identified periodontitis as a modifiable risk factor of rheumatoid arthritis (9), the treatment of which is strongly associated with a reduction of inflammatory parameters( (10), Peng et al., Gao et al.). As active periodontitis causes increased oxidative stress it may contribute to free radical production and all their detrimental effects: dementia, metabolic diseases, cancer, and vascular failure (11–13).

Immunological processes must not only be associated with pathology. Physiological bone turnover can be considered as an immunological process itself. Bone turnover is needed to cope with stress and aging (e.g. bruxism, resorption), bone healing (e.g. following extractions and implantology), and tooth movement (e.g. orthodontics) (Ma et al.).

All the mentioned processes function differently. However, the underlying metabolic pathways overlap at key points: the activation or inhibition of osteoblasts, osteocytes, and osteoclasts by diverse mediators which result in anabolic and catabolic bone metabolism.

Even if these key points were deciphered someday, the understanding of immunological processes of the maxillofacial bone would still pose challenges as new questions emerge which can – again – be demonstrated in the example of MRONJ. The disease was first attributed to the mechanism of action of bisphosphonates which either act as an ATP analogon (nitrogen-free) or infringe on the metabolism of HMG-CoA (nitrogen-containing) (14). Then it was found that denosumab was able to induce osteonecrosis by acting as a decoy receptor on the RANK – ligand (15). In the meantime, other (mainly antineoplastic) drugs have been discovered that can cause osteonecrosis either in isolation or in combination with chemotherapeutic regimens (16). Those pathomechanisms were not easy to investigate and have not been fully understood to date. But this realm continues to evolve. The new antiosteoporotic antibody romosozumab has now been associated with MRONJ development (17). Romosozumab blocks the effects of sclerostin, a mediator that reduces osteoblastic activity in bone homeostasis and has been identified as a central actor in the pathogenesis of osteoporosis (18). How can that be related to the pathophysiology of MRONJ caused by bisphosphonates and antiresorptives? Of course by deciphering the underlying immunological process.

The editors would like to highlight the importance of research on immunological processes of maxillofacial bone pathology through these introductory words and encourage readers (researchers and clinicians) to get involved with this Research Topic. As the mentioned examples showed, research in this field is a role model for the success of translational medicine efforts.

## Author contributions

MT: Conceptualization, Writing – original draft, Writing – review & editing.
